# Artificial Intelligence for Plant Gene Regulatory Networks

**DOI:** 10.3390/ijms27198819

**Published:** 2026-10-02

**Authors:** Haopeng Yu

**Affiliations:** 1 Sainsbury Laboratory, University of Cambridge, Bateman Street, Cambridge CB2 1LR, UK; haopeng.yu@jic.ac.uk; 2 Department of Cell and Developmental Biology, John Innes Centre, Norwich Research Park, Norwich NR4 7UH, UK

**Keywords:** gene regulatory network (GRN), plant transcription factor, artificial intelligence, single-cell multi-omics, foundation model, cis-regulatory element

## Abstract

Gene regulatory networks (GRNs) are the maps that plant biologists most want to draw, with arrows pointing from transcription factors to the genes they control, explaining how plants develop and respond to the environment. Since experimental validation of even one arrow can take years, and a single plant genome harbours tens of millions of candidate TF–target pairs, this gap has driven the development of numerous computational methods for GRN inference. Here I review three generations of computational approaches to plant GRN inference and measure them against a five-level ladder of evidence, including statistical association, conditional dependence, physical binding, functional regulation and causal circuits. Classical statistics and machine learning have simplified complex gene expression data into clear, prioritised predictions. Techniques like single-cell and multi-omics methods help uncover cell type-specific networks based on chromatin accessibility and binding motifs. Now, deep learning can interpret cis-regulatory grammar directly from genome sequences, and foundation models trained on plant genomes and millions of cells hold exciting potential to share regulatory insights across different species. The most promising route forward is a lab-in-the-loop division of labour in which foundation models serve not as oracles but as hypothesis engines, collapsing fifty million candidate TF–target pairs into the few hundred edges most worth the bench’s time, with each round of validation sharpening the next round of prediction.

## 1. Introduction

Gene regulatory networks (GRNs) play a central role in how plant biologists explain plant development and environmental responses at the molecular level. Whether the question is how a root senses nitrogen, how a meristem decides to flower, or how a crop survives drought, these processes are often described as networks, with transcription factors (TFs) directing “arrows” towards the genes they regulate, known as GRNs [1,2]. Those arrows carry the regulatory direction and explanatory weight. They nominate candidate genes, prioritise editing targets, and turn a list of differentially expressed genes into a mechanistic story. A GRN is, in this sense, the object plant biology most wants and most frequently draws.

Establishing all of those “arrows” experimentally, however, is a formidable undertaking. To demonstrate that a single TF regulates a single target gene in a plant, one must ordinarily build a transgenic series for that TF, CRISPR knockout alleles alongside overexpression lines, and then read out the transcriptional consequences of the perturbation, a process that routinely consumes several years [3,4]. In addition, specific plant TFs generally belong to gene families whose members act redundantly, so plant mutants must be assembled before any phenotype becomes visible [5,6]. Moreover, a typical plant genome encodes several thousand TFs and tens of thousands of genes, creating a space of millions of possible TF–target pairs [7,8]. Validating edges one experiment at a time therefore cannot keep pace with the size of the network, and the field has increasingly turned to genome-wide data that capture many regulatory relationships at once.

Over the past two decades, our capacity to generate gene regulatory network data has grown spectacularly. Public repositories hold expression profiles for tens of thousands of plant samples. Single-cell atlases now resolve transcription at cellular resolution across development, and in maize the same resolution has been achieved for chromatin accessibility [9,10,11]. Most distinctively, a genome-scale in vitro cistrome built by DAP-seq across several hundred *Arabidopsis* transcription factors provides experimentally measured binding preferences [8,12]. Yet these data do not reveal regulatory arrows on their own, and extracting them has driven the development of computational approaches to GRN inference.

Historically, computational approaches have undergone a series of transitions. The first generation applied classical statistics and machine learning (ML), from correlation and mutual information to tree-ensemble regression, to bulk expression compendia [13]. The second generation embraced single-cell and multi-omic data, coupling expression with chromatin accessibility to resolve cell type-specific networks [14]. The third and current generation brings deep learning (DL) and foundation models (FMs): neural networks that read regulatory ‘grammar’ directly from DNA sequence, and transformers pretrained on millions of cells that promise to transfer regulatory knowledge across species [15]. Each generation has strengthened a partnership in which computation surveys the genome-wide space of candidate edges and experiments are reserved for the few that an inference method ranks highest [16].

This review first walks through how a GRN is built from expression data, step by step, using a familiar plant biology example to illustrate each stage. It then traces three generations of computational approaches, from classical statistics and machine learning through single-cell and multi-omic integration to deep learning and foundation models, and evaluates them against five levels of claim that an edge can carry, from bare statistical association to a validated causal circuit. This review aims to make computational biology methods of GRN inference legible to plant biologists and to suggest that AI foundation models offer plant GRN inference a promising route from correlative analysis to causal explanation.

## 2. GRN Inference from Scratch

Imagine you are studying how *Arabidopsis* roots respond to nitrogen. After treating plants with nitrogen and profiling their roots by RNA-seq, you find that thousands of genes change expression. You wonder, ‘Which transcription factors orchestrate this response, and which genes does each of them control?’ The answer takes the form of a gene regulatory network (GRN): a map in which each node is a gene and each arrow points from a TF to a gene it regulates. Yet the *Arabidopsis* genome encodes about 2000 TFs and 28,000 genes [7], giving more than fifty million possible TF–target pairs, far too many to test experimentally one knockout at a time, so you decide to infer the network computationally from the expression data you have already collected.

Inference begins with an expression matrix, in which each row is a gene, each column is a sample, and each entry records how strongly that gene is expressed in that sample. In this experiment, the samples might be roots harvested at a series of time points after nitrogen supply, from minutes to hours, with replicates at each point. The founding intuition of the field is ‘guilt by association’: if a TF activates a target, the two should rise and fall together across samples [17]. For every TF–gene pair, you therefore compute a measure of statistical dependency, either Pearson correlation or mutual information (a more general statistic that also captures nonlinear relationships), and retain the pairs that score above a threshold [18]. The result is a co-expression network, and tools such as WGCNA can further organise it into ‘modules’ of genes that share an expression pattern and are therefore likely to serve related functions [19]. Co-expression is informative but symmetric, that is, it cannot tell which gene is the regulator and which is the target, nor whether two genes are connected directly or merely respond to a common upstream signal.

To sharpen these associations into directed edges, the next generation of methods reframes network inference as a prediction problem. GENIE3, the best performer in the DREAM5 community benchmark [16,20,21], asks a simple question for each gene in the genome: how well can this gene’s expression be predicted from the expression of all TFs? It trains a random forest model, a machine learning model that is an ensemble of decision trees, for every target gene, and the algorithm reports how much each TF contributes to the prediction [13]. TFs with high importance scores become the putative regulators of that gene. Repeating this across all genes yields a genome-wide, directed network in which every edge carries a confidence score, allowing you to focus on the top-ranked fraction rather than all fifty million possibilities.

These edges, however, are still statistical patterns, so the next step is to filter them against independent biological evidence of binding. The logic is simple: if TF X truly regulates gene Y, X’s binding site should occur in Y’s gene body, most likely in the promoter. You therefore keep an edge only when the TF’s binding motif is present in the target’s regulatory region. Better still, you can use experimentally mapped binding sites, such as those in the *Arabidopsis* DAP-seq cistrome [8], rather than predicted motifs alone. This motif-filtering strategy underlies SCENIC in the single-cell field [22] and plant tools such as MINI-EX [23]. By leveraging these tools, you can clearly visualise a network comprising hundreds of TFs and thousands of targets, with each arrow supported by both co-expression and binding evidence and each ranked by confidence.

The final phase uses the network as a hypothesis-generating machine. Highly connected ‘hub’ TFs become candidate master regulators of the nitrogen response, and the top-ranked edges become shortlists for experimental validation, for example, by knocking out the TF and testing whether predicted targets respond, or by inducibly activating it and recording the transcriptomic consequences [20]. Confirmed edges can then feedback as prior knowledge for the next round of inference.

## 3. The Claim Behind the Arrow

**Figure 1:**
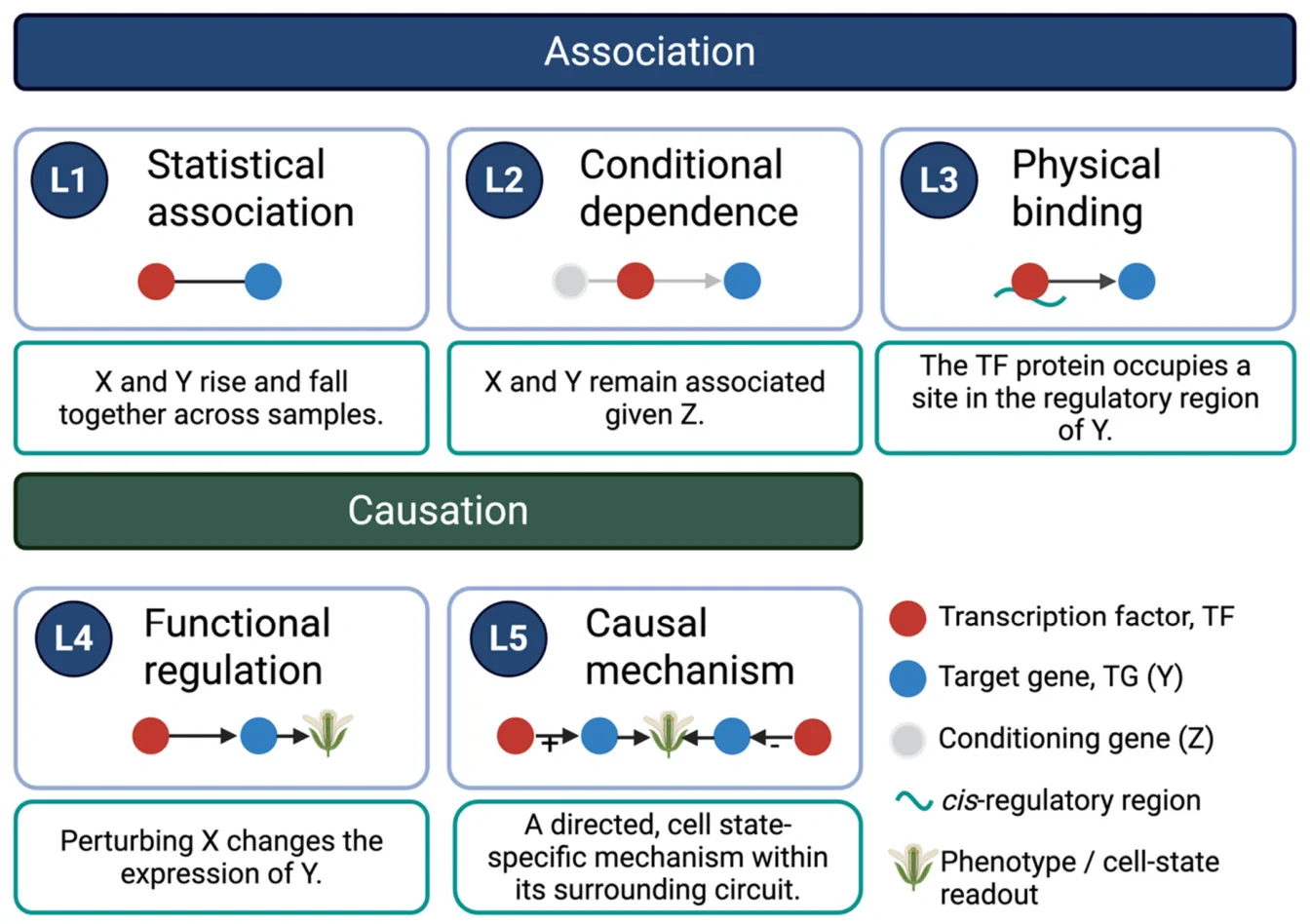
Five levels of evidence behind a regulatory edge. An edge “TF X → gene Y” can assert five progressively stronger propositions. Statistical association (L1), conditional dependence (L2) and physical binding (L3) remain within association; functional regulation (L4) and causal mechanism (L5) cross into causation. Symbols are defined in the key; the grey arrow at L2 marks an association explained away by Z, and “+” and “−” at L5 denote activation and repression. Created in BioRender (https://biorender.com/fyz63b3).

Although inferring “arrow” (the edge within the GRN) is the central task of GRN reconstruction, the term ‘edge’ denotes at least five distinct propositions, each established by a different kind of experiment (Figure 1 and Table 1). At the first level, an edge asserts only statistical association: gene X and gene Y rise and fall together, as measured by correlation. At the next level, it asserts conditional dependence: X and Y stay associated even after accounting for other genes.

Level 2 deserves a word more, because it is where most of the practical work of network inference happens. The most common false positive in co-expression analysis is the indirect edge: if X activates an intermediate regulator Z and Z in turn activates Y, then X and Y co-vary strongly, although X never acts on Y, and an upstream signal that drives both will do the same. Conditioning asks whether the dependence between X and Y survives once Z is known; if it disappears, the edge is explained away as indirect. This is what partial correlation does for linear dependence, what the data-processing inequality does in ARACNe by removing the weakest edge of every fully connected triplet [24], and what regression methods such as GENIE3 approximate by scoring each TF in the presence of all the others [13]. The claim is stronger than correlation, but it remains a statement about transcript distributions rather than about mechanisms.

At the third level, it asserts physical binding: the TF protein occupies a site near the gene, as measured by ChIP-seq, CUT&Tag, and DAP-seq [25,26,27]. At the fourth, it asserts functional regulation: perturbing X changes the expression of Y, which is what a knockout or an inducible activation assay measure. Only at the fifth level does the edge assert what a biologist usually means by ‘X regulates Y’: a directed, context-specific causal mechanism operating in a defined cell state.

**Table 1:** Five claims of an edge in the gene regulatory network.

Level	Edge Meaning	The Claim	Assay	Position in Plants
1	Statistical association	X and Y co-vary across samples or cells	Bulk and single-cell RNA-seq	Abundant across many species
2	Conditional dependence	X and Y remain associated after accounting for other genes	Large expression compendia	Achievable in model species
3	Physical binding	The TF protein occupies a site near Y	ChIP-seq, CUT&Tag, DAP-seq [25,26,27]	DAP-seq cistrome for hundreds of *Arabidopsis* TFs in vitro [8]
4	Functional regulation	Perturbing X changes the expression of Y	Knockout; inducible activation [28]	One TF per experiment; low throughput
5	Causal circuit	A directed, signed, cell state-specific mechanism	Pooled single-cell CRISPR screens	Effectively absent

Therefore, expression-based inference methods estimate the first two levels. The ‘gold standards’ they are scored against, that is, ChIP-derived catalogues, motif databases, and curated interactions, encode the third. However, the question the biologist wants answered sits at the fourth or fifth. The next generation of GRN inference must bridge them, moving beyond association and binding toward genuine biological causality.

## 4. Statistical Methods for GRN Inference

**Figure 2:**
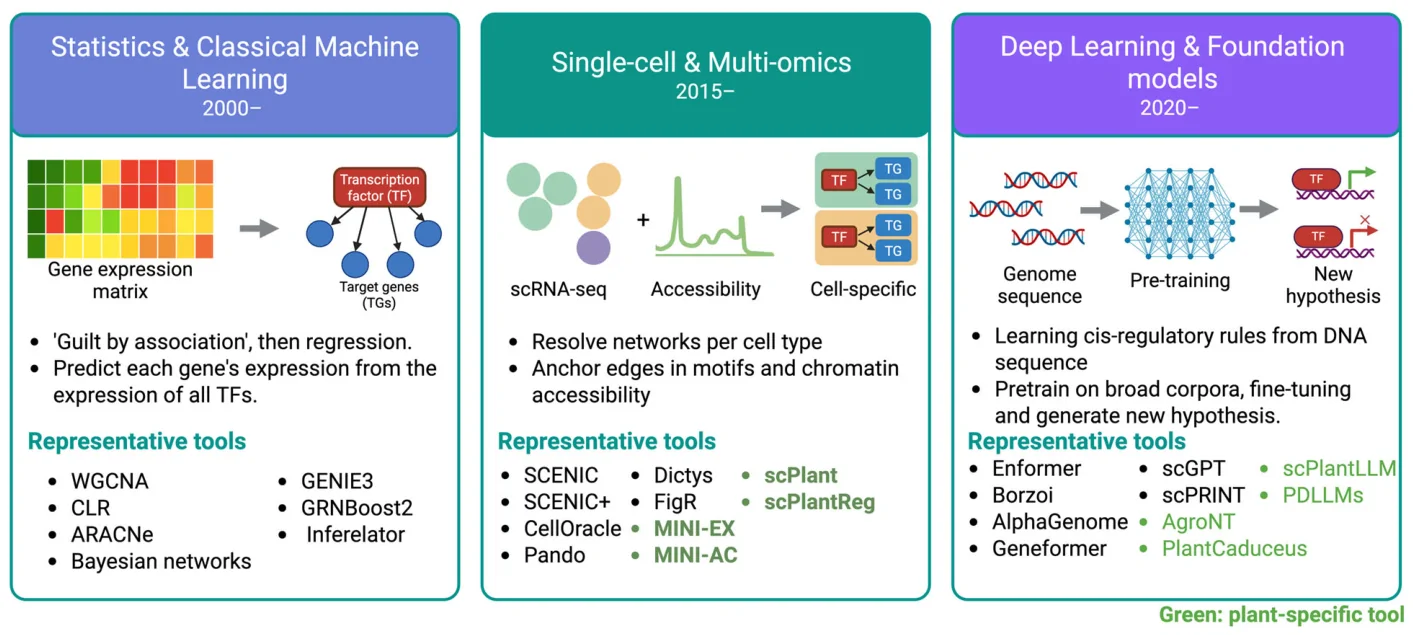
Three generations of GRN inference. Each generation changed the input data, the modelling idea and the strength of the edge claim it can support. Grey arrows indicate the analysis workflow. In the gene expression matrix, rows are genes and columns are samples, with colours from green to red indicating low to high expression. Coloured circles denote individual cells of different types; the green arrow and red cross denote a locus predicted to be active or inactive after in silico perturbation. Tool names in green are plant-specific methods/tools/models. Created in BioRender (https://biorender.com/5nwww0r).

The first generation of computational approaches to GRN inference applies classical statistics to bulk expression datasets (Figure 2, left panel), and they remain the most widely used tools in plant biology today. Their starting point is the ‘guilt by association’ logic. If a TF regulates a target, the two genes should co-vary across samples [17]. The simplest implementation computes the Pearson correlation for every gene pair and retains the pairs above a threshold. WGCNA, the first level of GRN inference, is a canonical tool in this family [19]. It extends the idea by grouping correlated genes into modules that share an expression pattern, within which highly connected hub genes become candidate regulators [19].

Information-theoretic methods overcome correlation’s limitation of detecting only linear relationships. Mutual information is a more general measure of statistical dependence that also captures nonlinear patterns, such as a TF that activates its target only above a concentration threshold. CLR (context likelihood of relatedness) strengthens this measure by scoring each TF–gene pair against the background distribution of all pairs involving either gene, which suppresses spurious associations [18]. ARACNe (algorithm for the reconstruction of accurate cellular networks) goes further and prunes likely indirect edges. For every fully connected triplet of genes, it removes the weakest edge, on the reasoning that if X regulates Y and Y regulates Z, the apparent X–Z association is probably indirect [24]. This pruning moves the analysis towards level-2 GRN inference.

Probabilistic graphical models make the field’s causal ambition explicit. A Bayesian network learns a directed graph together with a probability model of how each gene depends on its regulators, so its edges carry direction by construction [29]. The price of this ambition is high, learning the structure is computationally demanding, the number of samples required grows quickly with the number of genes, and the framework requires the graph to contain no cycles, a condition that real regulatory circuits, rich in feedback and autoregulation, do not satisfy. Taken together, the statistical methods established the foundations of the field, but they left two problems open: correlation and mutual information are symmetric and cannot orient an edge, while Bayesian networks can orient edges but do not scale to genomes with thousands of TFs. 

## 5. Machine Learning Predicts GRNs Based on TF Expression

Instead of asking whether two genes co-vary, machine learning-based methods ask how well each gene’s expression can be predicted from the expression of all TFs. GENIE3 implements this idea with random forests, training one model per target gene and converting the importance of each TF in the prediction into a directed, ranked edge [13]. Because each gene is modelled independently, the approach scales to whole genomes, accommodates feedback loops that Bayesian networks must exclude, and captures nonlinear and combinatorial effects that pairwise statistics miss [13]. GRNBoost2 accelerates the same design with gradient boosting, which makes it practical for single-cell datasets of tens of thousands of cells, and it serves as the inference engine inside SCENIC [22,30].

The other machine learning methods improve inference by adding biological knowledge rather than modelling power. Module networks infer regulators of whole modules rather than of single genes, borrowing statistical strength across co-expressed genes [31]. The Inferelator combines regression with prior knowledge of TF–target interactions and with estimates of TF activity derived from those priors [32]. Evaluations consistently find that such prior-informed methods outperform methods relying on expression alone [33,34].

## 6. The Single-Cell and Multi-Omic Data for GRNs

Single-cell transcriptomics resolves each cell type separately, captures rare cell types and developmental intermediates, and turns a study of a few dozen samples into a study of tens of thousands of cells [35]. For network inference, this means the question can change from ‘which TFs regulate this gene in the root’ to ‘which TFs regulate this gene in the root epidermis, at this developmental stage’.

Plant single-cell resources have grown quickly. The *Arabidopsis* root has been mapped at high resolution with developmental annotation [9], the maize single-cell cis-regulatory atlas provides the reference chromatin accessibility resource in plants [11], a rice atlas integrating data from multiple species now enables the study of cis-regulatory evolution [36], scPlantDB aggregates annotated plant cells across dozens of species [10], and the Plant Cell Atlas consortium has set out the community’s long-term goals [37]. Most inference tools from this era descend from a template established by SCENIC, inferring co-expression modules with a tree-ensemble regressor, keeping only modules whose members are enriched for the binding motif of the candidate regulator and scoring the activity of the resulting ‘regulon’ in every individual cell [22,38]. Based on these methods, it is feasible to obtain cell-specific, development-related TF regulatory rules.

Multi-omic profiling extends this design by measuring chromatin accessibility in the same cells or tissues as expression. SCENIC+ implements this idea by linking TFs through enhancers to their target genes [39]. CellOracle uses accessibility-constrained networks to simulate TF perturbations in silico and project the resulting cell-state shifts [40]. Dictys models regulation along developmental continua using TF footprinting [41]. Pando, notably, validated its multiome-derived predictions with pooled CRISPR screens in brain organoids [42]. FigR defines domains of regulatory chromatin that pair accessibility with expression [43]. scMTNI shares statistical strength across related cell types along a lineage tree [44]. Single-cell data also offer new handles on temporal direction, pseudotime orders cells along inferred developmental trajectories, and RNA velocity estimates each cell’s short-term future from the ratio of unspliced to spliced transcripts [45,46]. Both are increasingly used to orient network edges.

For plant readers, the most important development of this era is a small but growing set of tools built specifically for plants. MINI-EX performs integrative single-cell network inference with plant motif collections and cell-type specificity [23]. MINI-AC infers networks from bulk or single-cell chromatin accessibility using plant-specific motif and locus models [47]. scPlant provides a plant-tailored framework for single-cell analysis [48], and scPlantReg supports plant single-cell chromatin accessibility and cis-regulatory element analysis [49]. What distinguishes these tools is that they encode plant-specific decisions, motif collections drawn from plant databases, search windows calibrated to plant genome architecture, and annotations taken from plant atlases. In a decade, the field has moved from tissue-averaged co-expression to cell type-resolved networks in which expression and binding evidence come from the same experiment (Figure 2, Table 2).

## 7. Learning the Regulatory Code from Genome Sequence 

Deep learning entered gene regulation with a question none of the previous generations could ask: can gene expression be predicted from DNA sequence alone? These sequence-based models, together with the foundation models of Section 8, form the third generation of the field (Figure 2, right panel; Table 2). Where statistical methods start from an expression matrix, sequence-based models start from the genome itself and learn which promoter and enhancer features drive transcription. The lineage of these models runs from convolutional networks trained on chromatin features to Enformer, which combined convolution with transformer attention over roughly two hundred kilobases of sequence [50], to Borzoi, which predicts RNA-seq coverage directly [51], and to AlphaGenome, which extends the input context to a full megabase [52]. DeepSTARR predicted enhancer activity from sequence accurately enough to design synthetic enhancers de novo [53]. Sequence models provide independent evidence in network inference, linking TF to targets statistically through co-expression, and indicating if the target’s regulatory region contains features that support plausible regulation. 

Another deep learning method applies neural architectures to the inference GRN itself, asking whether a neural network can extract network structure from an expression matrix better than a random forest. CNNC treats the joint expression histogram of a gene pair as an image and classifies it with a convolutional network [54]. DeepSEM learns the network as the structure of a deep structural equation model [55] and a rapidly growing family of graph neural networks represents genes and their relationships explicitly [15]. 

## 8. Decoding the Gene Regulatory Code with Foundation Models

Foundation models are large-scale neural networks pretrained on broad and diverse genomic data, through which they acquire general-purpose representations of the genome that can then be fine-tuned to a wide range of downstream tasks with strong performance [65,66,67]. In gene regulation, this paradigm has produced two distinct families. DNA language models, also known as genomic foundation models (gFMs), are trained directly on genome sequences, allowing them to grasp the fundamental genetic patterns. On the other hand, single-cell foundation models are trained on millions of expression profiles, which helps them understand cellular behaviours. 

The DNA language models are an area where plant biology is unusually well served. AgroNT was pretrained on the genomes of 48 edible plant species and transfers to regulatory annotation tasks across crops [56]. PDLLMs is a group of DNA language models that have been tailored specifically to plant genomes [57], and PlantCaduceus, pretrained across sixteen angiosperm genomes at single-nucleotide resolution, identifies deleterious variants with severalfold greater sensitivity than conservation-based methods [58]. 

The single-cell foundation models are designed for network inference. Geneformer introduced transfer learning from large single-cell corpora and proposed in silico deletion, removing a gene from a cell’s input and observing the effect on the model’s representation, as a way to rank network-critical regulators [59]. scGPT extracts candidate gene networks from its attention maps [60]. scFoundation scaled the approach to one hundred million parameters [61]. Universal Cell Embedding achieved zero-shot cell representation across species [62], and scPRINT makes the most explicit network claim, deriving cell type-specific networks from attention and benchmarking them against interaction databases, ChIP-seq and Perturb-seq data [63]. In plant biology, scPlantLLM, a transformer pretrained on more than one million annotated *Arabidopsis* cells, reports zero-shot cell-type annotation accuracy of up to 0.91, transfers to maize and rice, and extracts gene networks and regulatory hubs from its attention scores [64].

## 9. Challenges, Validation and Benchmarking of GRN Inference in Plants

GRN inference in plants faces biological and technical challenges that are largely absent from the microbial and mammalian systems in which most methods were developed. Firstly, many crops are polyploid, and homoeologous gene copies may be expressed at different levels, diverge in function or act redundantly. As a result, an inferred edge can be assigned to the wrong copy or diluted across several copies [68,69]. Even in diploid species, plant TF families are large and partially redundant, which weakens the statistical signal of any single regulator [5,6]. In large, repeat-rich genomes such as maize, regulatory elements can lie tens of kilobases from their target genes, beyond the promoter-proximal windows assumed by most motif-based methods [70]. Extensive presence–absence variation further means that a network inferred in one accession may not transfer to another [71]. Plant transcriptomes are also strongly shaped by the diurnal cycle and by fluctuating environments, so samples collected at different times or under different conditions can produce associations that do not reflect direct regulation. Among the technical challenges, single-cell profiling requires enzymatic removal of the cell wall, and this treatment itself induces stress-responsive genes that can appear as spurious regulatory modules [72]. Outside *Arabidopsis*, rice and maize, most species also lack the genome annotation, binding data and curated motifs on which prior-based methods depend. 

Assessing the performance of GRN inference methods requires a benchmark comprising a reference network regarded as “ground truth”, a common set of input data, and agreed evaluation metrics reported relative to a random baseline. In non-plant systems, there are several generally used benchmarks. The DREAM5 challenge evaluated more than thirty methods on bacterial, yeast and simulated data and found that no single method performed best across all datasets. GENIE3 ranked highest overall, but an aggregated community prediction was more robust than any individual method [16]. BEELINE extended this assessment to single-cell data and showed that most methods approached random performance on realistic network topologies [73]. More recent benchmarks, including geneRNIB and the perturbation-based CausalBench, further showed that simple expression-based approaches such as Pearson correlation and GRNBoost2 often matched or exceeded more complex models [21,74].

In plants, method performance has so far been evaluated only within individual studies. In the *Arabidopsis* nitrogen response, a network inferred from time-series expression data was tested against TARGET-validated targets of 33 TFs. Its area under the precision–recall curve significantly exceeded that of random networks [20,75]. ConnecTF later integrated TARGET, ChIP-seq and DAP-seq interactions into a platform for scoring predicted networks [76]. An integrative *Arabidopsis* network that combined motif, chromatin, binding and expression data outperformed each of its input networks, and 75% of its predicted ROS regulators were supported by phenotypic assays [33]. In maize, co-expression networks built from more than 6000 RNA-seq samples were enriched for ChIP-seq targets and for genes that changed expression in TF mutants [77]. ChIP-seq of 104 maize TFs now provides the largest in vivo binding reference in a crop [78]. Together, these studies show that plant GRN predictions carry real biological signal. However, because each study used its own reference, tissue and metric, they cannot establish which method performs best.

Unfortunately, no standardised benchmark for plant GRN inference exists at present. The central obstacle is the absence of large-scale functional ground truth, because pooled perturbation of TFs remains technically difficult in plants owing to the cell wall, the bottleneck of transformation and regeneration, and extensive genetic redundancy [79,80]. Most available references, including in vitro DAP-seq peaks and the more than 10,000 regulome datasets compiled in ChIP-Hub [81], describe physical binding (Level 3 in Table 1) rather than functional regulation (Level 4). The same binding data are often used both as priors during inference and as gold standards during evaluation, which inflates apparent accuracy [22,23]. Reference data are also rarely matched to the tissue or condition of the prediction and remain concentrated in *Arabidopsis*, rice and maize. Polyploid crops add a further difficulty because redundant homoeologues make it unclear which gene copy an edge refers to [69]. Finally, evaluation criteria vary widely between studies. Plant single-cell tools have been evaluated mainly by their recovery of known cell-type regulators [23], and plant foundation models mainly by cell-type annotation [64], rather than by the accuracy of individual edges.

## 10. The Road from Association to Explanation

Over two decades, computational biology has produced an abundance of algorithms for GRN inference, and measured against the five levels of claim. Correlation and mutual information establish statistical association (Level 1); regression ensembles sharpen it into ranked, directed prediction (Level 2); and motif filtering and multi-omic integration anchor the surviving edges in physical binding (Level 3). Three generations of computational biology methods have climbed only three rungs of the ladder, and the reasons remain in the dataset.

A first ceiling is set by what a transcriptome can reveal about a regulator at all. Inference treats the mRNA abundance of a TF as a proxy for its activity. However, many plant regulators, including the DELLA, JAZ and Aux/IAA families, are controlled by hormone-triggered, proteasome-mediated degradation, so their protein abundance and activity can change independently of, or even in the opposite direction to, their transcript levels. The DREAM5 consortium reached the same conclusion empirically, finding that TF and target mRNA levels are correlated in *E. coli* but not in *S. cerevisiae* [16]. These same regulators also lack a canonical DNA-binding domain and act by binding partner TFs such as PIFs, MYC2 and ARFs, so binding-based evidence cannot place them in the network either. Compounding the problem, TFs are typically expressed at low levels and are therefore among the first genes lost to shallow sampling. Beyond deeper sequencing, this problem can currently be addressed in two ways. One is to measure a different layer, using DAP-seq binding and ATAC-seq accessibility to establish whether a TF is positioned to act [8,47]. The other is to infer TF activity from the coherent behaviour of its target set, an approach that is far more robust to missing values [22]. MINI-EX combines the latter two strategies and additionally retains a regulon whenever the TF is detected in at least 10% of the cells of a cluster, falling back to TF-family motif enrichment where a TF lacks its own model [23].

The other ceiling of the field is set less by modelling power than by the kind of data the models are given. In mammalian systems, pooled single-cell CRISPR screens read out the transcriptomic consequences of hundreds of knockouts in a single experiment, and their arrival has already changed practice. Pando’s multiome-derived network was tested and refined against precisely such a screen [42], and the most recent benchmarks use large-scale perturbation data as the ground truth itself [21]. In plants, this level of the ladder is effectively absent, held back by the cell wall, by the bottleneck of transformation and regeneration, and by the genetic redundancy that hides single-gene phenotypes [79,80]. This gap matters most for regulators that neither bind DNA nor report their activity through their transcripts, for which perturbation offers the only causal evidence; degradation-resistant alleles such as *gai* and *axr2*/*axr3* have long provided exactly this for DELLA and Aux/IAA proteins, but only one gene at a time. On the bright side, technological progress has never stalled. Transient systems such as TARGET already deliver TF perturbation and transcriptomic readouts within days rather than years [28], inducible activation time courses have mapped the temporal logic of nitrogen signalling, and protoplast platforms bring pooled delivery within reach [82]. Assembling these into a plant Perturb-seq, even a modest one covering a few hundred TFs in a single tissue, would be doubly transformative.

These advances should not be mistaken for causal inference. At the level of individual edges, agreement between predictions and experiments remains modest. In the *Arabidopsis* nitrogen network, the area under the precision–recall curve was 0.24, compared with 0.19 for random networks, and roughly two in three edges retained in the final network lacked support [75]. Large-scale perturbation benchmarks in other systems point to the same gap. Methods that exploit interventional data do not outperform those that use observational data alone [21]. None of five single-cell foundation models, including scGPT, Geneformer and UCE, predicted the transcriptomic effects of genetic perturbations better than simple linear baselines [83]. The in silico perturbations of Geneformer and CellOracle therefore describe shifts within a learned representation rather than the response of a living cell [40,59]. Likewise, the attention-derived networks of scGPT and scPlantLLM remain statements of association at Levels 1 and 2 [60,64]. In plants, none of these model-derived networks has yet been tested systematically against perturbation data. Until such tests are available, AI outputs should be treated as ranked hypotheses rather than causal conclusions, and a model should be judged by how often its top predictions are confirmed experimentally.

**Figure 3:**
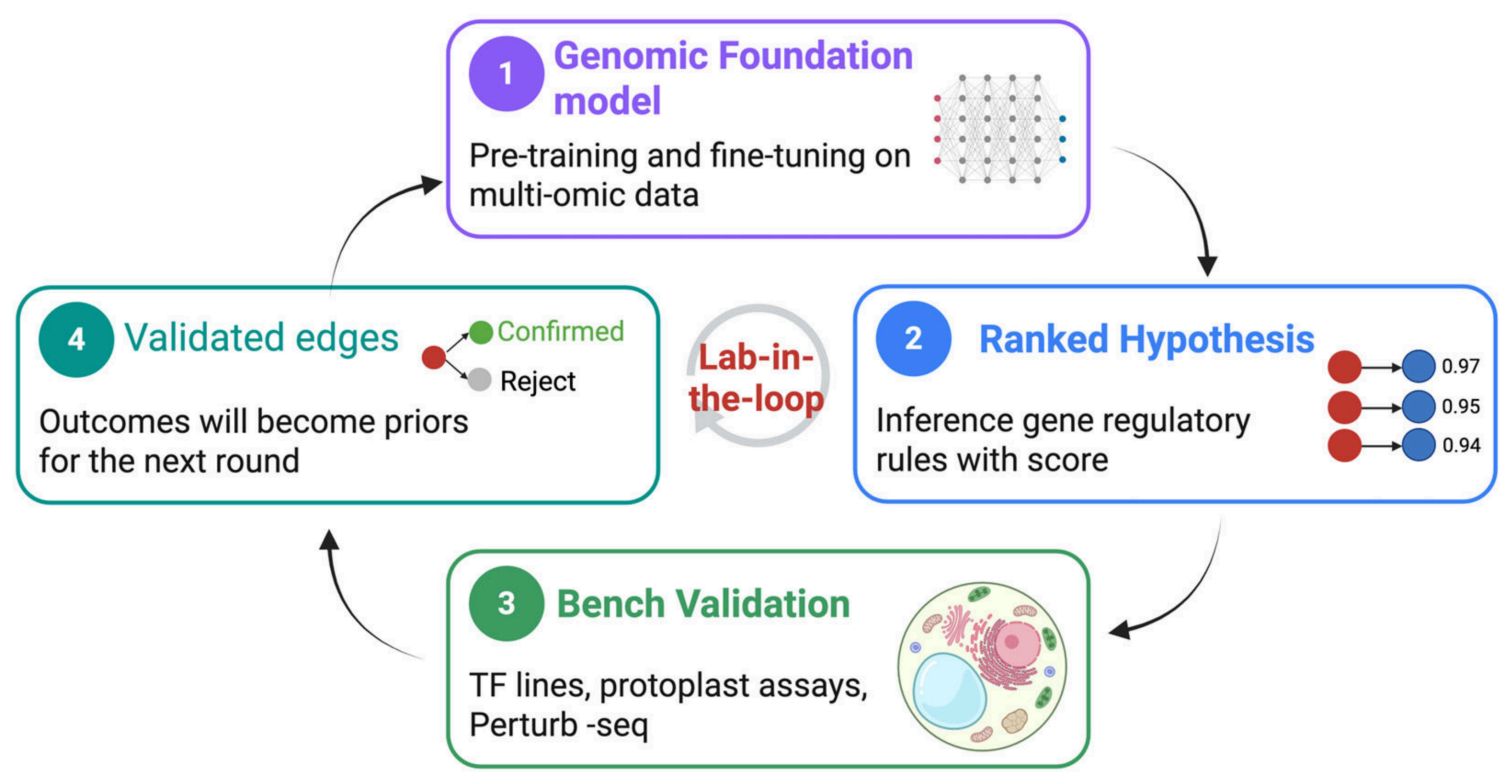
**A lab-in-the-loop design for plant GRN inference.** A multi-omics pretrained and fine-tuned genomic foundation model proposes confidence-ranked regulatory rules via in silico perturbation, whose most valuable and uncertain links are tested with inducible TF lines, protoplast assays, and eventually pooled plant Perturb-seq, with the results fed back to refine the model. Black arrows indicate the iterative cycle (steps 1–4). Red and blue circles denote TFs and target genes, respectively, with numbers indicating confidence scores; green and grey circles denote confirmed and rejected edges. Created in BioRender (https://biorender.com/1iabhi6).

This is where foundation models earn their place in the pipeline, not as oracles but as hypothesis engines. Geneformer’s in silico deletion removes a gene from a cell’s input and watches the representation shift [59]. CellOracle propagates a simulated knockout through an accessibility-constrained network and projects the resulting change of cell state [40]. scPRINT and scPlantLLM read candidate regulatory structure directly out of attention [63,64]. These methods generate ranked, genome-scale hypotheses, which are exactly what an expensive experiment needs. On the other hand, an FM-based method can be easily adapted to a lab-in-the-loop design. The model selects the edges where the truth is most uncertain and important. It then tests these with a quick assay or pooled screen. Based on the results, the model gets refined, and the next round of nominations becomes even better. It is a continuous process of learning and improving to make more accurate predictions. In such a lab-in-the-loop design (Figure 3), AI does not replace validation; it decides what is worth validating, collapsing fifty million candidate pairs into the few hundred that deserve the bench’s time.

For plant biologists, the choice of method should follow the data at hand. For bulk RNA-seq, GENIE3 or GRNBoost2 provides a robust ranked network [13,30,69], and WGCNA identifies co-expressed modules [19]. Where binding data exist, prior-informed methods such as the Inferelator, or filtering edges with the DAP-seq cistrome, improve specificity [26,32]. For scRNA-seq, MINI-EX is the natural starting point because it combines co-expression with plant motif collections [23]. For chromatin accessibility or multi-omic data, MINI-AC is tailored to plant genomes [47], whereas SCENIC+ and CellOracle require plant motif databases to be supplied [39,40]. Three pitfalls recur across all methods. Overfitting arises when thousands of TFs are fitted to few samples, and it can be detected by comparison with random networks and simple correlation baselines. Batch effects arise when data combine laboratories, genotypes or protoplasting protocols, and they should be corrected before inference. False positives, mostly indirect edges, are best limited by retaining top-ranked edges supported by independent binding evidence and by testing a subset experimentally. More tools will undoubtedly continue to appear, and no recommendation will remain current for long. Whichever method is chosen, I therefore recommend that researchers assemble a small ground truth for their own system, such as known regulator–target pairs or mutant transcriptomes. Scoring each tool against your reference, reveals whether its predictions are credible before any new edge is taken to the bench.

## 11. Conclusions

Few things in plant biology are as hard to establish as an arrow from a TF to its target gene, and twenty years of computational work have brought it steadily into focus. Classical statistics first tied the arrow to co-variation across samples. Machine learning then sharpened these associations into directed, ranked predictions. Single-cell profiling resolved the resulting networks cell type by cell type, revealing regulation that tissue averages had concealed. Multi-omics gave the surviving edges a physical basis in chromatin accessibility and TF binding. Sequence models added an independent line of evidence by reading regulatory grammar directly from DNA, and foundation models now promise to carry this knowledge across cells, tissues and species. Yet even these advances stop at the third level. They reveal which arrows are likely and where a TF could bind, but not whether perturbing the regulator actually changes the expression of its target. Closing this gap is therefore not a matter of choosing between computation and experiment, but of dividing the labour between them. Statistical and machine learning methods offer a rapid first survey of the genome-wide candidate space. Single-cell and multi-omic data place each candidate edge in its cellular context, after which sequence and foundation models condense this evidence into a short list of ranked, testable hypotheses. Perturbation experiments, transient today but increasingly pooled and scalable, act as the final arbiter, and every edge they confirm or reject is fed back into the next round of prediction. In this loop, an arrow is no longer merely drawn but earned.

## Figures and Tables

**Table 2:** Computational methods for gene regulatory network inference, organised by methodological generation.

Method	Year	Input Data	Algorithmic Principle	Output and Edge Claim
Generation 1 · Statistical methods (bulk expression)				
WGCNA [19]	2008	Bulk expression (microarray/RNA-seq)	Weighted correlation followed by hierarchical clustering into co-expression modules; intramodular hubs are nominated as regulators	Undirected modules; Level 1
CLR [18]	2007	Bulk expression compendia	Mutual information scored against the background distribution of all pairs involving either gene	Undirected, background-corrected pairs; Levels 1–2
ARACNe [24]	2006	Bulk expression	Mutual information combined with the data-processing inequality: the weakest edge of every fully connected triplet is removed	Undirected edges with indirect links pruned; Level 2
Bayesian networks [29]	2000	Bulk expression	Learns a directed acyclic graph together with a probability model of each gene given its regulators	Directed acyclic graph; Level 2
Generation 1 · Machine learning (regression-based)				
Module networks [31]	2003	Bulk expression	One shared regulatory program is learned per co-expressed module, borrowing statistical strength across genes	Regulators assigned to modules; Level 2
GENIE3 [13]	2010	Bulk or single-cell expression	One random-forest model per target gene; the importance of each TF in the prediction becomes an edge score	Directed, ranked, genome-wide; Level 2; top-ranked method in the DREAM5 benchmark [16]
GRNBoost2 [30]	2019	Single-cell expression	Gradient-boosting implementation of the GENIE3 design with an order-of-magnitude gain in runtime	Directed, ranked; Level 2; inference engine within SCENIC-type pipelines
Inferelator 3.0 [32]	2022	Expression with prior TF–target interactions	TF activities are estimated from priors, followed by regularised regression of targets on those activities	Directed, signed; Levels 2–3
Generation 2 · Single-cell and multi-omic methods				
SCENIC [22,38]	2017	scRNA-seq	GRNBoost co-expression modules are pruned by TF-motif enrichment; regulon activity is scored in every cell (AUCell)	Cell-type-resolved regulons; Level 3; the template followed by most plant single-cell tools
SCENIC+ [39]	2023	scRNA-seq + scATAC-seq (multiome)	Links TFs through candidate enhancers to their target genes	Enhancer-anchored eGRNs; Level 3
CellOracle [40]	2023	scRNA-seq + scATAC-seq	Accessibility-constrained regression; TF knockouts are simulated in silico and the resulting cell-state shifts are projected	Directed networks with perturbation simulations; Level 3 (Level 4 simulated)
Dictys [41]	2023	Multiome with developmental trajectories	TF footprinting in accessible chromatin, with networks modelled along developmental continua	Dynamic, context-specific networks; Level 3
Pando [42]	2023	Multiome (brain organoids)	Joint TF–region–gene regression across the multiome	Level 3, with predictions tested by pooled CRISPR screening (Level 4)
FigR [43]	2022	scRNA-seq + scATAC-seq	Pairs accessibility with expression within domains of regulatory chromatin (DORCs)	Regulatory domains and TF associations; Level 3
scMTNI [44]	2023	Multi-cell-type omics with a lineage tree	Multi-task learning shares statistical strength across related cell types along a lineage	Cell-type-specific networks; Levels 2–3
Generation 2 · Plant-specific single-cell tools				
MINI-EX [23]	2022	Plant scRNA-seq	GRNBoost inference combined with plant motif collections, cell-type specificity filtering and ranking	Ranked, cell-type-specific plant regulons; Level 3; applied in *Arabidopsis*, rice and maize
MINI-AC [47]	2024	Plant bulk or single-cell chromatin accessibility	Motif enrichment within accessible regions using plant-calibrated genome-wide and locus-based models	Accessibility-based networks; Level 3; applied in maize and *Arabidopsis*
scPlant [48]	2023	Plant scRNA-seq	Plant-tailored single-cell framework that includes a SCENIC-style GRN module	Plant regulons; Level 3; cross-species plant framework
scPlantReg [49]	2026	Plant scATAC-seq	Platform for single-cell accessibility and comparative cis-regulatory element analysis	cis-Regulatory maps supporting Level 3 evidence; multi-species plant resource
Generation 3 · Deep learning on genome sequence				
Enformer [50]	2021	DNA sequence (~200 kb context)	Convolutional layers with transformer attention predict expression and chromatin state from sequence alone	Sequence-encoded regulatory grammar; independent Level 3 evidence; trained on human and mouse genomes
Borzoi [51]	2025	DNA sequence	Predicts RNA-seq coverage directly from sequence as a unifying readout of regulation	Sequence → expression prediction; Level 3 support
AlphaGenome [52]	2025	DNA sequence (1 Mb context)	Unified multi-track model for regulatory variant-effect prediction	Variant effects across regulatory layers; Level 3 support
DeepSTARR [53]	2022	Sequence with STARR-seq enhancer activity	Convolutional network predicts quantitative enhancer activity and supports de novo enhancer design	Enhancer activity from sequence; Level 3 support; developed in Drosophila
Generation 3 · Deep learning applied directly to network inference				
CNNC [54]	2019	Paired-gene expression histograms	The joint expression histogram of a gene pair is treated as an image and classified with a convolutional network	Supervised edge classification; evidence level determined by the training labels
DeepSEM [55]	2021	scRNA-seq	Deep structural equation model in which the network is learned as the adjacency matrix of the model	Directed networks; Level 2
Graph neural networks [15]	2020s	Expression with prior networks represented as graphs	Genes and their relationships are represented explicitly in the graph structure; a rapidly growing method family	Output varies by architecture
Generation 3 · DNA language models (genomic foundation models)				
AgroNT [56]	2024	48 edible plant genomes	Transformer language model pretrained on plant genomes and fine-tuned for regulatory annotation across crops	cis-Regulatory element prediction; Level 3 support; plant-native foundation model
PDLLMs [57]	2025	Plant genomes	A family of DNA language models tailored to plant genomes	Multi-task plant genomic annotation
PlantCaduceus [58]	2025	16 angiosperm genomes	Bidirectional state-space model at single-nucleotide resolution with cross-species pretraining	Deleterious-variant and regulatory inference across species; severalfold higher sensitivity than conservation-based methods
Generation 3 · Single-cell foundation models				
Geneformer [59]	2023	~30 M human single-cell transcriptomes	Rank-value transformer; in silico deletion removes a gene from the input and the representational shift is measured	Context-specific candidate regulators; simulated Level 4; transfer-learning paradigm
scGPT [60]	2024	~33 M cells	Generative pretraining, with candidate gene networks extracted from attention maps	Candidate networks; Levels 1–2
scFoundation [61]	2024	~50 M profiles; 100 M parameters	Large-scale pretraining for a broad range of downstream single-cell tasks	Embeddings and perturbation prediction
UCE [62]	2026	Cross-species cell corpora	Zero-shot universal cell embedding across species	Cross-species cell representation; a route past plant data scarcity
scPRINT [63]	2025	~50 M cells	Pretraining with an explicit gene-network objective; attention-derived networks benchmarked against ChIP-seq and Perturb-seq data	Cell-type-specific networks; Levels 2–3; the most explicit network claim among single-cell foundation models
scPlantLLM [64]	2025	>1 M annotated *Arabidopsis* cells	Transformer for plant single-cell atlases providing zero-shot annotation, with networks and hubs derived from attention scores	Plant gene networks and regulatory hubs; Levels 1–2; transfers to maize and rice

## Data Availability

No new data were created or analyzed in this study. Data sharing is not applicable to this article.
